# *Bartonella henselae* Antibodies in Serum and Oral Fluid Specimens from Cats

**DOI:** 10.3390/pathogens10030329

**Published:** 2021-03-11

**Authors:** Alejandra Álvarez-Fernández, Marta Baxarias, David Prandi, Edward B. Breitschwerdt, Laia Solano-Gallego

**Affiliations:** 1 Departament de Medicina i Cirurgia Animals, Facultat de Veterinària, Universitat Autònoma de Barcelona, Bellaterra, 08193 Barcelona, Spain; Alejandra.alvarez@uab.cat (A.Á.-F.); Marta.baxarias@uab.cat (M.B.); david.prandi@uab.cat (D.P.); 2 Intracellular Pathogens Research Laboratory, Department of Clinical Sciences, Comparative Medicine Institute, College of Veterinary Medicine, North Carolina State University (NCSU), Raleigh, NC 27607, USA; ebbreits@ncsu.edu

**Keywords:** bartonellosis, feline, serology, immunofluorescence antibody assay, oral fluid

## Abstract

Cats are the primary reservoir host for *Bartonella henselae* *(B. henselae*), an etiological agent of human bartonellosis, including cat scratch disease. Although *Bartonella* DNA has been amplified from salivary swabs from cats, dogs and humans, we are not aware of studies investigating *Bartonella* antibodies in oral fluid (OF). Using inhouse and commercial immunofluorescence antibody assays (IFA), the objective of this study was to detect and compare antibodies against *B. henselae* in paired OF and serum specimens from cats. Specimens were collected from shelter and client-owned cats. For serum specimens, *B. henselae* seroreactivity was 78% for both the inhouse and commercial IFA assays and 56.8% for OF specimens. Comparing serum and OF specimens, there was moderate Kappa agreement (Cohen’s k = 0.434) for detection of *B. henselae* antibodies. Oral fluid antibodies were more likely measurable in cats with high *B. henselae* serum antibody titers when compared with low antibody titers. In conclusion, *B. henselae* OF IFA antibody measurements were less sensitive compared to serum IFA measurements of ≥1:64. Oral fluid antibodies were detected more often in cats with high *B. henselae* serum antibody titers. Therefore, OF antibodies, detectable by IFA, is of limited utility for epidemiological or diagnostic testing in cats.

## 1. Introduction

Cats are the primary reservoir for *B. henselae*, the etiological agent of human cat scratch disease, and a major cause of bartonellosis across animal species [1,2]. *Bartonella henselae* is considered a stealth pathogen, particularly in dogs and humans, where published data is expanding the spectrum of clinical manifestations that have been historically associated with this infection [3].

For clinicians and researchers, documentation of *Bartonella* infection in most animal species remains a challenge due to the lack of sensitive and/or specific testing modalities. Testing limitations apply to diagnostic assays used to evaluate cats, dogs and humans for indirect (antibody) or direct (culture, antigen, DNA) evidence of *Bartonella* spp. infections [4,5,6]. Despite a wide variety of currently available commercial and research testing modalities, a negative serological or direct detection test result does not guarantee the absence of this stealth pathogen [7,8,9]. The combination of a thorough clinical data analysis, including age, sex, animal and arthropod vector exposure history, physical examination findings, ancillary laboratory testing, in conjunction with indirect and direct diagnostic microbiological techniques is the most effective strategy for the diagnosis of *Bartonella* spp. infections [6,10,11].

The immunofluorescence antibody assay (IFA) is the most widely employed serological technique to measure *Bartonella* spp. IgM or IgG antibodies in patient sera [12]; however, Enzyme-Linked Immunosorbent Assays (ELISA) [13] and Western immunoblot assays are also commercially available [14]. Seronegativity is an important limitation during early acute infection and in a subset of animal and human patients during longstanding bloodstream infections [15,16]. In the context of direct detection, PCR is the most frequently used molecular assay to detect the presence of *Bartonella* spp. DNA in patient specimens, including whole blood, fresh or frozen tissue, lymph node aspirates and saliva [17,18]. Combining enrichment culture prior to PCR testing is a diagnostic strategy developed to optimize the documentation of “active” infection [19]. Moreover, low and potentially relapsing bacteremia rates may represent limiting factors for the sensitivity of PCR and enrichment blood culture/PCR techniques. Noninvasive specimens, such as saliva, have been analyzed using PCR to detect *Bartonella* spp. DNA in cats [20,21]. Oral fluids (OF) include whole saliva, fractionated saliva, gingival crevicular fluid, and dentinal tubular fluid [22]. Therefore, oral fluid samples include not only saliva but also crevicular and dentinal tubular fluids, which we refer to collectively as OF in this study. To our knowledge, OF has not been used for the detection of antibodies against *B. henselae* antigens in cats. Salivary IgG originates via gingival transudation from the capillary network below the oral mucosa [23]. Documentation of salivary IgG offers clinicians and researchers the possibility to obtain an alternative diagnostic specimen for the detection of *Bartonella*-specific antibodies. Saliva has been used previously as the diagnostic specimen for detecting antibodies against feline immunodeficiency virus (FIV) and feline leukemia virus (FeLV) in cats [24,25]. In general, it is substantially easier, less stressful, and less invasive to collect saliva than a blood sample from feline patients. In addition, it does not need much expertise. However, both venipuncture and saliva collection could be a challenge in some cats without sedation, particularly in the feral cat populations targeted in seroepidemiological studies [26]. The aim of this study was to detect and compare antibodies against *B. henselae* IFA antigens in paired serum and OF specimens from cats.

## 2. Results

### 2.1. Description of the Study Cats

All the cats were European domestic short hair. Sixty-seven of 118 (56.8%) were female and 51 (43.2%) were male cats. Their ages ranged from 4 months to 7 years, with a median age of 1.7 years. For statistical analyses, cats ≤ 2 years old were considered young cats and >2 years old were considered old cats. One hundred and sixteen of 118 (98.3%) were sexually intact at the time of entry into the study. Shelter and client-owned cats comprised 55.1% (65/118) and 44.9% (53/118), of the study population, respectively. Owned cats ranged in age from 5 months to 7 years, with a median age of 2.4 years. Shelter cats ranged in age from 4 month to 5 years, with a median age of 1.2 years. Shelter cats were more likely to be young cats (73.8%) when compared with old cats (37.7%) (Chi square test: X2 = 15.59, df = 1, *p* < 0.001). History of tick and flea infestations were reported in 56.8% (67/118) of the cats, while 19.5% (23/118) were flea-infested at the time of sampling (21 shelter cats and two client-owned cats). Shelter cats were more likely to have a history of tick and/or flea infestation (100%), when compared with client-owned cats (3.8%) (Fisher exact test, *p* < 0.001). Routine treatment with ectoparasiticides was only reported for 25 client-owned cats, 21.2% (25/118) of total cats. One hundred cats (84.7%) were assessed as clinically healthy, whereas 18 (15.3%) cats had clinical signs with gingivitis being the most frequent clinical sign found in nine sick cats (50%). 

### 2.2. Inhouse Versus Commercial IFA Testing of Cat Sera

**Figure 1:**
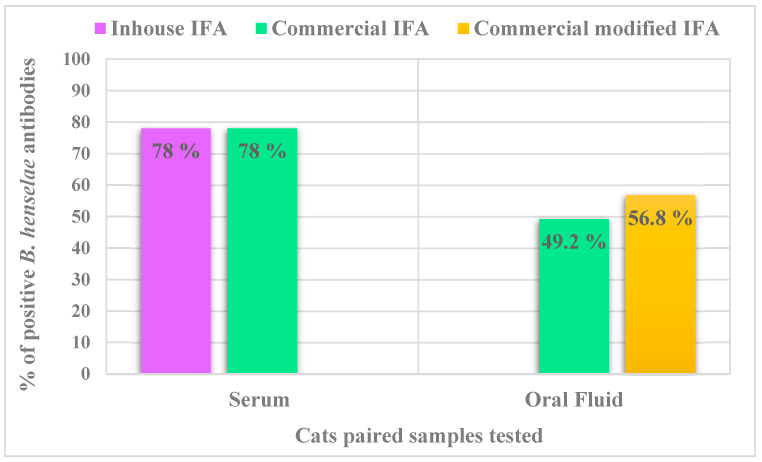
Comparison of the proportion of IFA positive results in serum and OF specimens for the detection of antibodies against *B. henselae* antigens. Abbreviations: IFA, immunofluorescence antibody assay.

Considering antibody titers ≥ 1:64, 78% (92/118) cats were *B. henselae* seroreactive when tested using both the inhouse and commercial IFA assays (Figure 1). The *B. henselae* serum IFA geometric mean titer was 1:2088 with minimum and maximum titers of 1:64 and 1:16,384, respectively. *Bartonella henselae* antibody titers ranged from 1:64 to 1:512 in 35.9% (33/92) and >512 in 64.1% (59/92) of the seroreactive cats.

When considering antibody titers ≥1:64 for the 118 cats in this study, Kappa agreement, was almost perfect when comparing the inhouse IFA and the commercial IFA kit (Cohen’s k = 1; 95% confident interval) (Table 1). 

**Figure 2:**
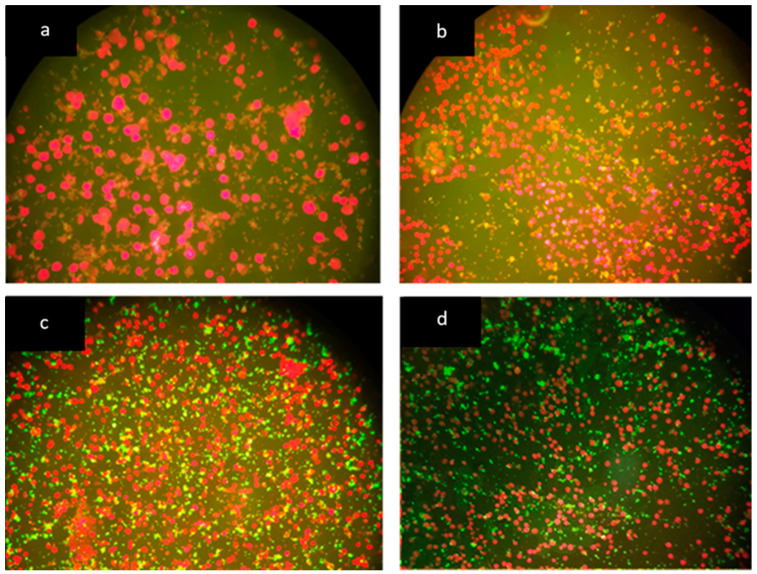
Visual subjective criteria for classification of *B. henselae* antibody concentration according to fluorescence intensity. (**a**) Negative, cells stain in red (400×), (**b**) negative/nonspecific fluorescence (200×), cells stain in red, (**c**) low positive (200×), and (**d**) high positive (200×). *Bartonella henselae* organism stain apple green in panels (**c**,**d**).

There was moderate Kappa agreement (Kappa = 0.587; 95% confidence interval) when comparing negative, low positive and high positive inhouse IFA antibody titers with the fluorescence intensity for the commercial IFA (Figure 2). Based upon comparative analysis, shelter cats were more likely to have serum *B. henselae* antibodies (100%) when compared with client-owned cats (50.9%) (Fisher exact test, *p* < 0.001) (Table 2). Furthermore, shelter cats were more likely to have high serum antibody titers (>1:512.) against *B. henselae* (69.3%) when compared with client-owned cats (26.4%) (Chi square test: X2 = 42.99, df = 2, *p* < 0.001). When age was considered, cats ≤ 2 years were more likely to be *B. henselae* seroreactive (98.5%) than cats >2 years (50%) of age (Fisher’s exact test: *p* = 0.001).

### 2.3. Commercial IFA in Serum and OF Samples

Using the commercial IFA, 78% (92/118) of cats were *B. henselae* seroreactive. All cats *B. henselae* seronegative in both IFA assays were also negative in the OF IFA assay (26/118). When seroreactivity results were categorized using specific fluorescence intensity as the criteria described in Figure 2, 22% (26/118) of cats were seronegative, 15.3% (18/118) were low positives and 62.7% (74/118) were high positives. Only 49.2% (58/118) of cat OF samples were *B. henselae* antibody positive prior to the sample concentration step. When comparing serum and OF samples, there was moderate Kappa agreement for detection of *B. henselae* antibodies, using the commercial IFA assay (Cohen’s k = 0.429; 95% confidence interval) (Table 1). Considering the end point inhouse IFA titer results, OF samples were more likely to be positive in cats with high *B. henselae* serum antibody titers (>1:512) (77.2%) when compared with low antibody titers (≤1:512) (22.8%) (Fisher’s exact test: P = 0.001). Comparisons between commercial serum and OF IFA results, when categorized as low positive or high positive based upon inhouse IFA antibody titers are provided in Table 3. Oral fluid antibodies were also more frequently found in young cats (≤2 years old) (64.7%), compared to older cats (>2 years old) (28%) (Chi square test: X2 = 18.87, df = 1, *p* < 0.001). The statistical comparison of the proportions of positive results based on clinical parameters in both serum and OF samples are listed in Table 2. Serum and OF antibody titers did not differ between sick cats with gingivitis and the rest of the sick and healthy cats. However, all sick cats with gingivitis were seroreactive and seven of nine cats with gingivitis were OF positives. Six gingivitis cats had serum antibody titers ≥1:512.

### 2.4. Modified Commercial IFA Testing of OF Specimens

Fifty-two of 60 available *B. henselae* antibody OF negative samples and 10 antibody OF positive samples were retested using the modified IFA. All previously positive IFA OF samples remained *B. henselae* positive, 9 of 52 previously IFA negative samples became IFA positive. In total 56.8% (67/118) of OF samples were *B. henselae* antibody reactive when tested by both IFA assays. Of the 92 *B. henselae* seroreactors, 67 (72.8%) were OF antibody positive. Comparison of the proportions of positive results in serum and OF using the two IFA assays are shown in Figure 1. There was moderate Kappa agreement for detection of *B. henselae* antibodies, when comparing total results obtained in serum and OF samples using the commercial (not modified) and modified IFA assays (Table 1, Cohen’s k = 0.541; 95% confidence interval).

## 3. Discussion

Although several studies have documented the presence of *Bartonella* spp. DNA in oral samples obtained from cats [20,27,28], to the best of our knowledge, studies reporting the presence of *B. henselae* antibodies in OF samples have not been published. Among the 92 *B. henselae* seroreactors in this study, only 67 (72.8%) were also OF antibody positive. In agreement with our findings, a previous study determined that total IgG was lower in oral samples when compared with serum samples [29]. To collect the OF samples, we used cotton swabs soaked with hypertonic NaCL 7.5% to increase oral transudation and salivation. Therefore, we obtained a higher volume of OF; however, it is possible that the OF antibody concentration was diluted by the saline soaking of the swabs. In a study investigating total IgG using ELISA, higher OF immunoglobulin concentrations were obtained using unstimulated samples (dry swabs) [29]. Unfortunately, it was not possible in the present study to collect OF samples with dry swabs, likely due to an inhibitory effect of the anesthetic drugs on the production of OF. In order to increase IFA OF sensitivity, we used the commercial IFA with modifications, which resulted in 9 out of 52 negative samples being positive. It is possible that detection of *B. henselae* antibodies in OF specimens could be improved by concentrating saliva samples and thereby increasing the overall and *B. henselae*-specific antibody concentrations. Consistent with this concept, a recent study focused on the IgG purification of human saliva in order to achieve a high similarity of IgG antibody profiles from blood and saliva for diagnostic testing purposes [30].

In the present study, there were no statistical differences in *B. henselae* seroreactivity among cats with gingivitis compared to the rest of all cats, which is in agreement with previous studies [31,32]. However, all sick cats with gingivostomatitis in this study were *B. henselae* seroreactive and the majority were OF positive as well. High antibody titers were reported in a previous study that established a relationship between oral lesions and *B. henselae* and *Bartonella clarridgeiae* antibodies [21]. The results of this study differ from studies involving FIV seropositive cats [26] and cats with chronic gingivostomatitis [29]. FIV seropositive cats had increased salivary IgG levels, but this result was partly attributable to the presence of oral inflammatory lesions as suggested by the higher ratios of salivary IgG than serum IgG in both FIV seronegative and seropositive cats with oral lesions compared to cats without oral lesions [26]. Similarly, cats with chronic gingivostomatitis had significantly higher salivary IgM and IgG, but significantly lower salivary IgA concentrations than healthy cats [33]. In contrast, higher salivary IgA and lower IgM and IgG were reported in healthy cats using ELISA and single radial immunodiffusion (SRID) assays [29]. In addition to the clinical status (healthy vs. sick for gingivostomatitis) of the cat, variable results in detection of antibodies may be due to the use of different diagnostic techniques, IFA and Western blot had also been used as immunoglobulin diagnostic techniques for OF [24]. In dogs, similar concentrations of anti-*Leishmania* IgG2 antibodies were found in serum and saliva measured by time-resolved immunofluorometric assays (TR-IFMAs), likely due to the high sensitivity of this assay [34].

Because of many factors, IFA assays could be considered subjective techniques, including the skills and experience of the technologist in results interpretation, antibody cross-reactions, and antigen sources used. These and other factors influence IFA sensitivity and specificity. In this study, we found identical seroreactivity results using inhouse and commercial *B. henselae* IFA assays. Furthermore, there was moderate agreement when comparing antibody intensity categories in the commercial assay to inhouse IFA antibody titers. Although it is necessary to develop more studies focused on the determination and measuring of antibodies in OF or saliva as a diagnostic technique, recently published OF optimization studies performed in other infections [24,29] could indicate that *B. henselae* OF could provide an efficient alternative to serum. Despite the low sensitivity of OF when compared with serum, OF noninvasive specimens could be an important resource for antibody detection procedures in cats and other animals from whom blood collection is challenging. In the present study, swabs were maintained in the mouth for 3−4 min but it is likely that with less time similar results will be obtained. Therefore, further OF collection and sample processing studies are needed to enhance the sensitivity of OF for *Bartonella* spp. antibody detection diagnosis in cats.

In this study, the very high *B. henselae* seroprevalence indicates that infection with *Bartonella* is common among shelter cats with high ectoparasite exposure in areas around Barcelona. Others studies involving cats from Spain have reported *Bartonella* spp. seroprevalence rates of 23.8% (Madrid) [35], 29.6% (Catalonia) [36], 35.3% (Catalonia) [37], and 71.4% (Catalonia and Mallorca Island) [38]. Seroprevalence differences between Spanish studies likely relate to the source of the cats, with higher risk being associated with stray and shelter cats because of extensive flea exposure and lack of prophylaxis. This study is in agreement with evolving evidence that cats living in the Mediterranean region have higher *Bartonella* spp. prevalence than others areas in Spain and Europe [4].

## 4. Materials and Methods

### 4.1. Cats

One hundred and eighteen cats from Barcelona province (Spain) were enrolled into the study between 2017 and 2019. A clinical questionnaire was completed for each cat including information about age, breed, sex, shelter/client-owned, health status (sick versus healthy), presence of fleas and /or ticks, or bites and the use of acaricide products. For all cats, a complete physical examination was performed by a veterinarian. In most cases (*n* = 110), blood specimens were obtained under general anesthesia during a routine neutering procedure.

### 4.2. Serum Specimens

For each cat at the time of enrollment, peripheral blood was collected by jugular or cephalic venipuncture, with 4–6 mL placed in serum tubes containing a clot accelerator and granule serum separator (Aquisel, Barcelona, Spain). These samples were centrifuged at 790× *g* for 10 min (Heraeus Labofuge 400R, Thermo Fisher Scientific, Waltham, MA, USA) to obtain the serum that was stored at −80 °C until used.

### 4.3. Oral Fluid Specimens

To obtain the OF, a swab (Ecouvillon PP, Dominique Dutscher, Bernolsheim, France) was soaked with hypertonic NaCL 7.5%. (B. Braun Melsungen AG, Melsungen, Germany) to obtain oral transudation and salivation. The swap was placed into the cat’s mouth between the gum and the inner mucosa of the upper or lower lip. To obtain the OF sample for antibody testing, the swab was maintained in that position for 3−4 min, after which the swab head was placed in an Eppendorf tube and centrifuged at 16000× *g* for 10 min (Eppendorf Centrifuge 5418, Merck KGaA, Darmstadt, Germany). OF samples were stored at −80 °C until tested.

### 4.4. Bartonella henselae Inhouse IFA Serological Testing

Initially, the 118 cat sera were tested at North Carolina State University, Intracellular Pathogens Research Laboratory using a previously inhouse *B. henselae* IFA assay for end point antibody titration [15,38] with some modifications and validated in dogs [7]. Briefly, to obtain antigens for IFA testing, *B. henselae* SA2+ (feline origin Missy 95 FO-099)*,* was passed from agar grown cultures into DH82 (a continuous canine histiocytic cell line) cultures. Heavily infected cell cultures were spotted onto 30-well Teflon-coated slides (Cel-Line/Thermo Scientific), air-dried, acetone-fixed, and stored frozen. Serum samples were diluted in phosphate buffered saline (PBS) solution containing 1% normal goat serum, 0.05% Tween-20 and 0.5% powdered nonfat dry milk (BioRad, Hercules, CA, USA) to block non-specific antigen binding sites. Patient sera were further tested with 2-fold dilutions out to a final dilution of 1:16,384 and 10 µL of every serum dilution was applied per well. The slides were incubated for 30 min at 37 °C and washed with PBS under moderate agitation for another 30 min. Once slides were dry, 10 µL of fluorescein-conjugated goat IgG anti-cat (MP biomedicals, Irvine, CA, USA) at dilution of 1:100 was added into each well. The slides were incubated for another 30 min at 37 °C in the dark to protect the photosensitive conjugate. The washing procedure described above was repeated adding a few drops of Tween 20. After the last washing procedure, some drops of mounting medium (Vectashield, Burlingame, CA, USA) were added on the cover slips. The slides were evaluated using a fluorescence microscope (OPTIKA Fluo B-383, OptikaItaly, Ponteranica, Italy) at 200× and 400× magnification and each well was compared to the fluorescence pattern seen in the positive and negative controls. To avoid confusion with possible nonspecific binding found at low dilutions, a cutoff of 1:64 was selected as a seroreactive antibody titer [38]. Cats were considered seronegative at titers <1:64. For statistical comparisons, cats were categorized as low antibody positive for titers between 1:64 and 1:512 and high antibody positive for titers >1:512. Experimental infection studies in cats have documented that the geometric mean of maximum antibody titer peak was ≥1:512 [15,39]. Moreover, previous studies in dogs and humans have classified antibody titers of ≥1:512 as high positives [40,41].

### 4.5. Bartonella henselae Commercial IFA Paired Serological and OF Testing

An indirect immunofluorescence assay (MegaFLUO *B. henselae*, Diagnostik Megacor, Hörbranz, Austria) for the detection of IgG antibody against *B. henselae* antigens was performed on paired sera and OF samples. Immunofluorescence antibody assay was performed for all cats included in this study at the Autonomous University of Barcelona. The serum samples were diluted to 1:64 with PBS while OF samples were not diluted. Twenty microliters of every paired sample, diluted serum and not diluted OF from the same cat, were applied in different wells in the same slide. The slides were incubated for 30 min at 37 °C. After that, a washing procedure was performed. The slides were washed twice with PBS for 5 min and once with distilled water. After the washing procedure described, 15 μL of FLUO FITC anti-cat IgG conjugate was added into each well. The slides were incubated for another 30 min at 37 °C in the dark to protect the photosensitive conjugate. The washing procedure described above was repeated. After the second washing procedure, some drops of mounting medium were added on the cover slips. The slides were evaluated using a fluorescence microscope (Leica DM6000 B; Leica Microsystems, Wetzlar, Germany) at 200× and 400× magnification and each well was compared to the fluorescence pattern seen in the positive and negative controls. All samples were examined by two different investigators to avoid observer error. Serum samples that were not fluorescent at a 1:64 titer were considered seronegative according to the manufacturers’ instructions and as previously described [42,43]. To compare OF versus serum antibody reactivity, the intensity of the specific organism (*B. henselae*) fluorescence observed was used as subjective criteria for classifying antibody concentration into negative, low positive and high positive (Figure 2). Due to economic restrictions, endpoint antibody titration was not performed with *B. henselae* commercial IFA whereas serum antibody titration was determined by the inhouse IFA.

### 4.6. Modified B. henselae Commercial IFA OF Testing

For the 52 of the 60 OF samples that were *B. henselae*-antibody negative, a modified IFA assay was performed. There was inadequate volume to retest eight OF negative samples. The modified IFA procedure was performed for 10 previously OF positive samples and 52 OF negative samples. The objective of this modification was to determine if *B. henselae* OF antibodies could be detected by adding double the volume of the OF. Thus the modified commercial IFA used 40 µL of OF instead 20 µL. Indirect immunofluorescence assay (MegaFLUO *B. henselae*, Diagnostik Megacor, Hörbranz, Austria) for the detection of specific IgG antibody against *B. henselae* antigens was performed by adding a second OF sample incubation step. Following the protocol described above, after the first washing procedure, another 20 µL of nondiluted OF sample was applied in the same well of first incubation, after which the slide was incubated again for 30 min at 37 °C. The rest of the commercial IFA protocol was followed without modification and the results were read as described above using classification criteria shown in Figure 2.

### 4.7. Statistical Analysis

The cumulative data collected from each cat, as well as the *B. henselae* antibody detection, in paired serum and OF samples were evaluated statistically. Frequency analysis for age, sex, stray/client-owned, ectoparasites, healthy status and the different IFA assays results was assessed. Comparative analysis of categorical data was performed by using chi-square test or Fisher’s exact test. The kappa (k) statistic was used to assess the degree of (interrater) agreement between end point inhouse IFA results and commercial IFA results classified using the subjective criteria described in Figure 2. Kappa was used also to assess the degree of agreement between commercial IFA used to test serum and OF samples. The following interpretation of the k statistic was: k < 0 poor, k = 0–0.2 slight, k = 0.21–0.4 fair, k = 0.41–0.6 moderate, k = 0.61–0.8 substantial, k = 0.81–1 almost perfect agreement. A significant association was deduced when *p* < 0.05. The statistical analysis was performed using the R program i386 version 3.6.1 (R Development Core Team) (R Foundation for Statistical Computing, Vienna, Austria) and the Deducer R program version 1.7–16 (Deducer: a data analysis GUI for R) (R Foundation for Statistical Computing company, Vienna, Austria) for Windows software.

## 5. Conclusions

In conclusion, OF antibodies were more likely to be detected in cats with high *B. henselae* serum antibody titers. When compared with sera in this study, measuring *B. henselae* OF antibodies by IFA testing was less sensitive. Therefore, without further successful optimization of *Bartonella* spp. antibody detection, OF specimens do not appear to be an adequately sensitive epidemiological or diagnostic assay for testing cats. In the context of test reproducibility, the inhouse and commercial IFA assays generated identical seroreactive and seronegative results for the 118 cats in this study.

## Figures and Tables

**Table 1:** Comparison of IFA agreement in serum and OF samples.

Test Pair	κ ± SE	κ Interpretation ^a^
Inhouse IFA serum versus commercial IFA serum	1 ± 0.000	Almost perfect agreement
Commercial IFA serum versus commercial IFA OF	0.429 ± 0.068	Moderate agreement
Commercial IFA serum versus commercial modified IFA OF	0.541 ± 0.072	Moderate agreement

^a^ The interpretation for each κ value is shown in the final column according to the following scale: ≤0, no agreement; 0.01–0.20, none to slight; 0.21–0.40, fair; 0.41–0.60, moderate; 0.61–0.80, substantial; and 0.81–1.00, almost perfect agreement. Abbreviations: IFA, immunofluorescence antibody assay; OF, oral fluid; κ, Cohen’s kappa value; SE, standard error.

**Table 2:** Comparison of demographic and clinical variables among cats based upon the proportion of commercial IFA *B. henselae* seroreactors in serum and OF.

Specimens	Variables														
	Lifestyle			Age			Sex			Clinical Status			Ectoparasites Presence		
	Client Client-Owned (*n* = 53)	Shelter (*n* = 65)	*p* Value	≤2 Years (*n* = 68)	>2 Years (*n* = 50)	*p* Value	Females (*n* = 67)	Males (*n* = 51)	*p* Value	Healthy (*n* = 100)	Sick (*n* = 18)	*p* Value	Yes (*n* = 23)	No (*n* = 95)	*p* Value
Serum	50.9%	100%	<0.001 *	98.5%	50%	<0.001 *	79.1%	76.5%	0.732 *	75%	94.4%	0.118 **	100%	72.6%	0.004 **
OF	20.7%	72.3%	<0.001 *	64.7%	28%	<0.001 *	52.2%	45.1%	0.442 *	45%	72.2%	0.086 *	78.3%	42.1%	0.002 **

IFA: immunofluorescence antibody assay. *n*: number. OF: oral fluid. * Chi-square test; ** Fisher’s exact test; a significant association was deduced when *p* < 0.05.

**Table 3:** Comparison between commercial *B. henselae* IFA results in serum and OF, when categorized as low positive and high positive to the serum inhouse antibody IFA titer results.

	Number of Positive Cats			
Serum Inhouse IFA Antibody Titers	Commercial IFA			
	Serum		OF (Total) *	
	Low Positive	High Positive	Low Positive	High Positive
Low positive 1:64—1:512 (*n* = 33)	11	22	8	5
High positive >512 (*n* = 59)	8	51	19	26
Total (*n* = 92)	19	73	27	31

* Total OF results including commercial modified IFA. Abbreviations: IFA, immunofluorescence antibody assay; n, number; OF, oral fluid.

## Data Availability

The data presented in this study are available on request from the corresponding author.

## Abbreviations

BAPGM	*Bartonella* Alpha-Proteobacteria Growth Medium
*B. henselae*	*Bartonella henselae*
ELISA	Enzyme-linked immunosorbent assay
FIV	Feline immunodeficiency virus
FeLV	Feline leukemia virus
IFA	Immunofluorescence antibody assay
IgG	Immunoglobulin G
IgM	Immunoglobulin M
k	Cohen’s Kappa value
NCSU	North Carolina State University
OF	Oral fluid
PBS	Phosphate buffered saline
PCR	Polymerase chain reaction
SE	Standard error
SRID	Single radial immunodiffusion
TR-IFMAs	Time-resolved immunofluorometric assays
